# Metastatic Axillary Cutaneous Squamous Cell Carcinoma in an African-American Female: A Rare Case

**DOI:** 10.7759/cureus.68892

**Published:** 2024-09-07

**Authors:** Shahriar Sharif, Biura Markarian, Diego Marin

**Affiliations:** 1 Internal Medicine, Hospital Corporation of America (HCA) Florida Westside Hospital, Plantation, USA; 2 Osteopathic Medicine, Nova Southeastern University Dr. Kiran C. Patel College of Osteopathic Medicine, Fort Lauderdale, USA; 3 Pulmonary and Critical Care, Boca Raton Regional Hospital, Boca Raton, USA

**Keywords:** adrenal gland metastasis, african-american female, axillary abscess, cscc, cutaneous squamous cell carcinoma, metastatic axillary squamous cell carcinoma, periaortic lymph node metastasis

## Abstract

Cutaneous squamous cell carcinoma (cSCC) is a common skin cancer, typically affecting older White males in sun-exposed areas, and metastasis is rare. We present a unique case of a 46-year-old obese African-American woman with a recurrent, deep abscess in her left axilla. It was initially treated with several incision and drainage procedures and antibiotics. Despite multiple interventions, the abscess recurred with severe pain and drainage. Subsequent biopsies revealed a high-grade malignant neoplasm, later confirmed as poorly differentiated cSCC with primary metastases to the lungs and secondary metastases to the adrenal glands and periaortic lymph nodes. Immunohistochemical staining supported the diagnosis. The patient's atypical presentation, including her race, a non-sun-exposed site, and younger age, highlights the need for vigilance in diagnosing cSCC in atypical cases. This case underscores the importance of early consideration of cSCC in differential diagnoses for persistent or recurrent abscesses, which can facilitate timely treatment, potentially preventing extensive metastasis and improving patient outcomes.

## Introduction

Cutaneous squamous cell carcinoma (cSCC) is a prevalent skin cancer, with approximately 1 million cases reported annually in the United States, primarily affecting older males in sun-exposed regions. Major risk factors include fair skin, increased UV exposure, and immunosuppression [1-3]. The majority of cases occur on sun-exposed areas such as the head, neck, and arm extensor surfaces [3]. cSCC is rare in African-American populations due to increased melanin levels providing protection against UV light. When it does occur, however, it tends to affect non-sun-exposed areas, particularly the lower extremities [3-4]. Etiologies for this presentation include persistent ulcers, non-healing wounds, burn wounds, human papillomavirus (HPV) infection, and exposure to ionizing radiation, as well as immunosuppressive conditions and rare inherited conditions such as albinism and xeroderma pigmentosum [1,3,5].

Studies on cSCC reveal an overall metastasis rate of 1.2-5%, with factors such as larger tumor diameter, increased tumor depth, extent beyond subcutaneous fat, perineural invasion, and poor differentiation increasing metastatic potential [1,6]. Approximately 80% of cSCC metastases spread to regional lymph nodes, but metastasis can also occur in the lungs, liver, brain, and bones [6].

This report presents a unique case of cSCC in a 46-year-old African-American female where the primary tumor was masked by a recurrent axillary abscess, leading to extensive metastases in the lungs, adrenal glands, and periaortic lymph nodes. This case is notable for its rarity in affecting a non-sun-exposed area, the absence of pertinent history, the relatively young age of onset, the female sex, and the African-American race.

## Case presentation

A 46-year-old, obese African-American woman presented to our hospital’s emergency department (ED) with a recurring, foul-smelling, deep abscess in her left axilla. Five months earlier, she had undergone an incision and drainage (I&D) procedure for an abscess in the left axilla at another institution and reported serosanguineous fluid drainage afterward with mild pain. The patient had reported to our hospital’s ED two months after the initial I&D, with a complex, indurated, deep abscess in the left axilla after pain and swelling in the area for two weeks. Our hospital had performed an I&D procedure, and the fluid collection had a clear brownish fluid that, once drained, revealed a cavity measuring at least 10 cm. No suspicious lesions were noted. She was tested for HIV, which came back non-reactive. She was discharged on linezolid and ciprofloxacin prior to the wound culture results, which later made *Staphylococcus* epidermidis sensitive to linezolid.

One month later, the patient presented back to our ED complaining of drainage and discomfort in her left axilla. She denied any past medical history, including any immunocompromised states; however, she also reported that she had not sought medical care for several years. She denied having any lesions or abscesses in the other axilla, groin, or perianal areas and denied a history of any scars, burns, or ulcers on the affected area prior. She reported a 10-pack-year cigarette smoking history and had quit smoking less than two months prior to the initial visit. She denied past and present drug and alcohol use. She also denied recent travel. Labs were significant for a white blood cell (WBC) count of 13,200/mm^3^ (range: 4500-11,000/mm^3^) and platelet count of 445,000/mm^3^ (range: 150,000-400,000/mm^3^). All other labs were within normal limits. She was admitted to the hospital for management of the axillary abscess. The surgical team performed another I&D. She was discharged with Omnicef and Percocet, and later wound cultures grew *Escherichia coli* sensitive to Omnicef.

The patient returned to our service after a couple of weeks with the same complaint of purulent drainage from her left axilla, severe, constant pain, and now abdominal pain, nausea, non-bloody vomiting, and diarrhea for several days. She reported she was non-compliant with the antibiotics as they hurt her stomach. Vitals were significant for a temperature of 100.9°F and a heart rate of 114 bpm. Labs were significant for a WBC count of 23,000/mm^3^ (range: 4500-11,000/mm^3^) and platelet count of 414,000/mm^3^ (range: 150,000-400,000/mm^3^). All other labs and vitals were within normal limits. She was admitted to the hospital and ended up staying for nine days for further evaluation of her persistent condition and to obtain imaging as described below.

**Figure 1:**
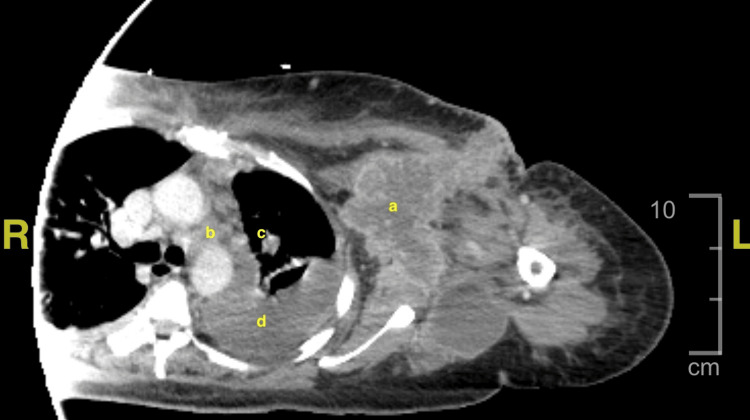
Computed tomography (CT) scan of the chest with contrast demonstrating axillary abscess, soft tissue mass, pulmonary nodule, and pleural effusion (a) left axillary abscess measuring 7.9 x 9.0 x 9.5 cm, (b) left hilar soft tissue mass measuring 3.6 cm, (c) left upper lobe pulmonary nodule measuring 1.6 cm, and (d) left pleural effusion

**Figure 2:**
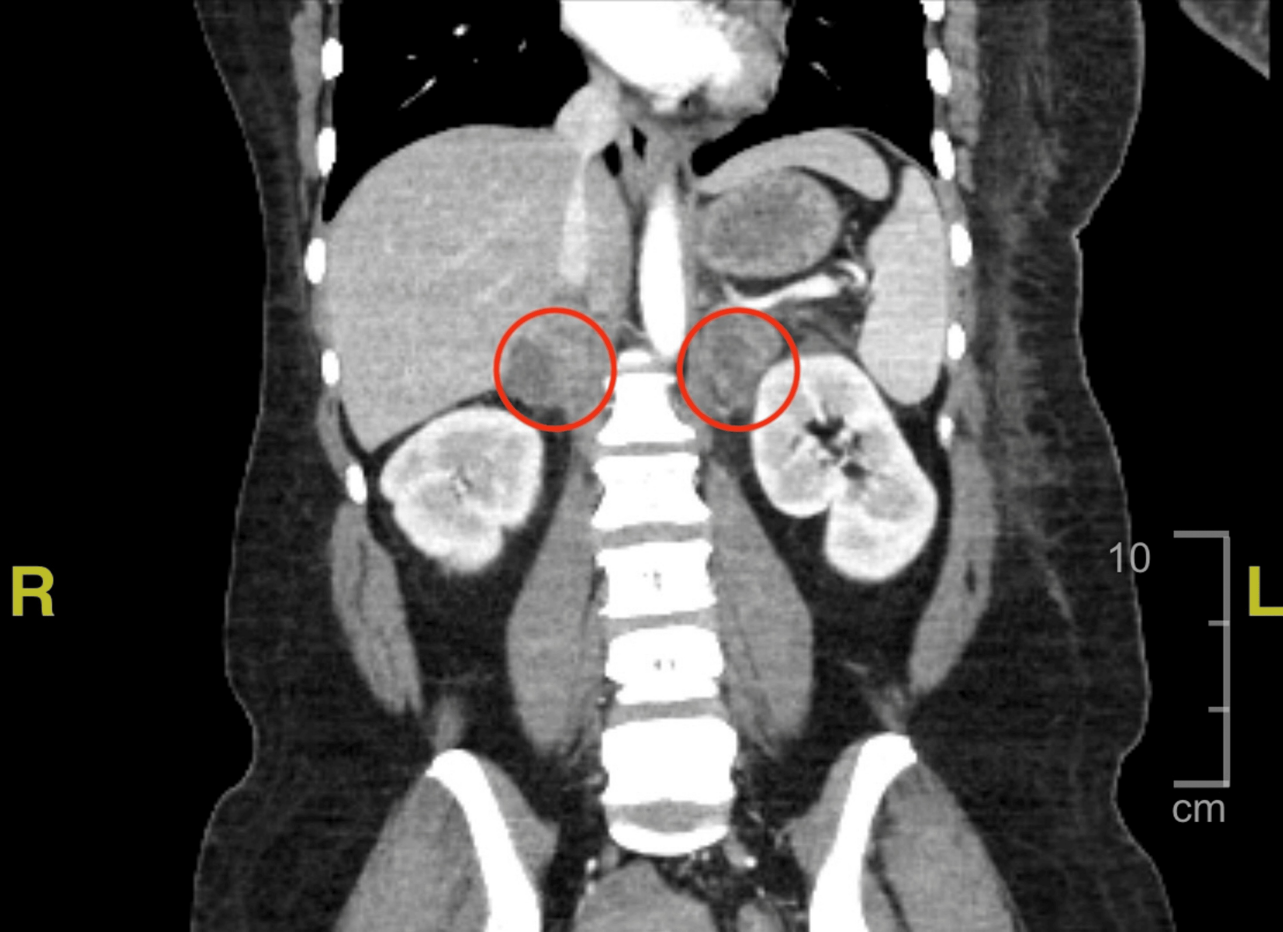
Computed tomography (CT) of the abdomen/pelvis with contrast demonstrating enlarged adrenal glands bilaterally Left red circle: right adrenal gland measuring 5.3 x 2.5 cm. Right red circle: left adrenal gland measuring 5.6 x 2.9 cm

**Figure 3:**
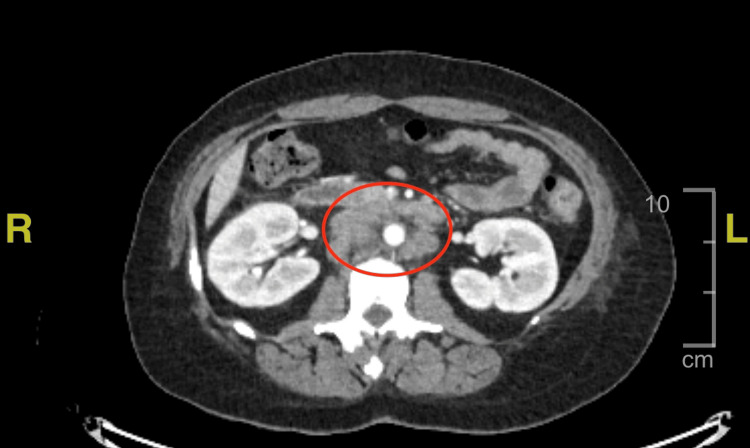
Computed tomography (CT) scan of the abdomen/pelvis with contrast demonstrating numerous enlarged periaortic lymph nodes Red oval: multiple periaortic lymph nodes of varying sizes

A chest computed tomography (CT) scan with contrast revealed an approximately 7.9 x 9.0 x 9.5 cm left axillary abscess with anterolateral dermal extension, as well as a left hilar soft tissue mass (3.6 cm), left upper lobe pulmonary nodule (1.6 cm), and left pleural effusion (Figure 1). On a CT scan of the abdomen and pelvis with contrast, the adrenal glands were enlarged bilaterally (right 5.3 x 2.5 cm, left 5.6 x 2.9 cm), and numerous enlarged periaortic lymph nodes were seen (Figures 2-3). These combined findings raised suspicion for metastatic disease.

The left axilla abscess cavity, left posterior axilla skin edge, and lung nodule were biopsied. Immunohistochemical staining of all three revealed a diagnosis of poorly differentiated cSCC. All three biopsies also stained positive for pancytokeratin, cytokeratin 5/6 (CK5/6), and cytokeratin 7 (CK7). All three biopsies also showed positive nuclear staining for P40. E-cadherin stained positive in the axilla and skin biopsies. The P16 marker was negative in the axilla and skin biopsies. Thyroid transcription factor-1 (TTF-1) stained negative on the lung biopsy.

## Discussion

cSCC is a common type of skin cancer that tends to affect fair-skinned individuals with prolonged sun exposure, especially older males [1]. In this case, we presented a 46-year-old African-American female with cSCC that manifested as a recurrent axillary abscess with eventual metastasis to her lungs, adrenal glands, and periaortic lymph nodes.

The immunohistochemical staining revealed a diagnosis of poorly differentiated cSCC. Pancytokeratin is a highly reliable marker for certain high-risk cSCC types [7]. CK5/6 is a marker for basal cell, squamous cell, and transitional cell carcinomas, and CK7 is a marker seen specifically in poorly differentiated subsets of cSCC [8-9]. E-cadherin has also been detected in high-risk cSCCs [7]. Furthermore, P40 is not only a cSCC marker, but it has also been shown to be more specific than two long-established markers (p63 and MNF116) [1,10]. As the P16 marker was negative, we ruled out HPV-related pathology [11]. Finally, TTF-1 is a highly sensitive and specific marker for diagnosing primary lung adenocarcinomas, and as it was negative in the lung biopsy, we ruled out lung malignancy as the primary diagnosis [12].

Chronic wounds, such as ulcers or abscesses in our patient’s case, can occasionally give rise to cSCC. The main cause is a Marjolin ulcer, a rare, aggressive skin cancer, most commonly squamous cell carcinoma, that arises from such chronically inflamed tissue as wounds, burn scars, and pressure sores [13]. They can develop in any age group and race, with men affected more often than women [13]. The time between initial injury and cancer development is usually long, averaging about 31 years [13]. Our patient’s initial presentation at the time of her diagnosis of cSCC lasted around seven months. While she denied any history of scars, burns, or ulcers on the affected area, it was unclear how long this abscess had been present prior to her initial presentation.

Factors that increase the risk of malignant transformation of such chronic wounds include delayed wound healing, infection, fragile tissue, and poor immune response [13]. These ulcers are more common in individuals of low socioeconomic status and in developing countries, often due to delayed medical attention [13]. As our patient had not sought medical care for several years, it is possible that this applies to her case and there could have been a delay in her wound healing. This could have led to a malignant transformation of her wound, especially given the aggressive nature and rapid progression of her disease.

Our patient was not a surgical candidate at the time of her diagnosis due to metastasis. The National Comprehensive Cancer Network recommends cytotoxic therapy with cisplatin, carboplatin, 5-fluorouracil, or a combination regimen with these drugs, with the latest update recommending carboplatin and paclitaxel combined with radiation therapy [14].

cSCCs are greatly responsive to immunotherapies and help avoid the toxicity of chemotherapy, and our patient opted for this route. Cemiplimab, pembrolizumab, and nivolumab are checkpoint inhibitors, specifically anti-programmed death-1 (anti-PD-1) antibodies that are approved for treating metastatic cSCC [14]. Cemiplimab was the first to be approved by the Food and Drug Administration for advanced cSCC and had an overall response rate (ORR) of 50% in Phase I and 47% in Phase II clinical trials [15]. Pembrolizumab is also an anti-PD-1 antibody approved for metastatic and/or recurrent cSCC with a 50% ORR in Phase II trials [15].

Despite urges from our team for the patient to follow up with the hematology/oncology doctor’s clinic as soon as possible for either chemotherapy or immunotherapy, the patient and her family insisted on a second opinion after the official diagnosis was revealed and explained to them. One week later, the patient presented back to our hospital with shortness of breath and was coded three times in the intensive care unit, with the final code resulting in the decision to stop resuscitation.

## Conclusions

This case report highlights a rare presentation of cSCC in a 46-year-old African-American woman with a recurrent, deep abscess in her left axilla. As cSCC typically presents in older white males and in sun-exposed areas, this patient’s race, age, and presentation in non-sun-exposed sites were rare and took a few hospital visits for the underlying cSCC to be identified. Unfortunately, at the time of the official diagnosis, there were already metastases, and our patient succumbed to the disease. The delay in diagnosis and subsequent metastasis highlights the importance of considering cSCC in differential diagnoses for persistent abscesses, regardless of typical risk factors or presentation sites. This case emphasizes the importance of thoroughly investigating unusual or non-healing wounds to detect potential malignancies early, which is crucial for improving outcomes and reducing mortality. It also serves as a reminder to maintain a high index of suspicion for cSCC in atypical presentations, facilitating timely diagnosis and more effective management.
